# A Genome-Wide Scan Divulges Key Loci Involved in Resistance to Aphids (*Aphis craccivora*) in Cowpea (*Vigna unguiculata*)

**DOI:** 10.3390/genes13112002

**Published:** 2022-11-01

**Authors:** Patrick Obia Ongom, Abou Togola, Christian Fatokun, Ousmane Boukar

**Affiliations:** 1 International Institute of Tropical Agriculture (IITA), PMB 3112, Kano 700223, Kano State, Nigeria; 2 International Institute of Tropical Agriculture (IITA), PMB 5320, Ibadan 200284, Oyo State, Nigeria

**Keywords:** aphid resistance, candidate gene, cowpea (*Vigna unguiculata* L. Walp.), genome-wide association study (GWAS), mini-core, single nucleotide polymorphisms (SNPs)

## Abstract

Cowpea aphids (*Aphis craccivora* Koch) double as a direct damaging pest and a virus vector to cowpea, threatening the economic yield of the crop. Given the multiple ecotypes, different alleles have been implicated in aphid resistance, necessitating the identification of key genes involved. The present study implemented a genome-wide scan using 365 cowpea mini-core accessions to decipher loci involved in resistance to aphid ecotype from Kano, Nigeria. Accessions were artificially inoculated with *A. craccivora* in insect-proof cages and damage severity assessed at 21 days after infestation. Significant phenotypic differences based on aphid damage severity were registered among the accessions. Skewed phenotypic distributions were depicted in the population, suggesting the involvement of major genes in the control of resistance. A genome-wide scan identified three major regions on chromosomes Vu10, Vu08 and Vu02, and two minor ones on chromosomes Vu01 and Vu06, that were significantly associated with aphid resistance. These regions harbored several genes, out of which, five viz *Vigun01g233100.1*, *Vigun02g088900.1*, *Vigun06g224900.1*, *Vigun08g030200.1* and *Vigun10g031100.1* were the most proximal to the peak single nucleotide polymorphisms (SNPs) positions. These genes are expressed under stress signaling, mechanical wounding and insect feeding. The uncovered loci contribute towards establishing a marker-assisted breeding platform and building durable resistance against aphids in cowpea.

## 1. Introduction

Cowpea (*Vigna unguiculata* L. Walp), a diploid (2n = 22) with genome size of 640.6 Mbp [1], is a key legume in the dry and hot regions of the world, particularly in Sub-Saharan Africa, where it is a prime source of protein. The bulk of world cowpea production and consumption is in the savanna regions of West Africa [2], where Nigeria leads with an annual production of about 3.65 million metric tons [3]. Productivity of cowpea in this region and other parts of the world is hindered by numerous insect pests, cowpea aphids, A. craccivora Koch (Homoptera: Aphididae), being one of the most important pests, causing severe economic damage during the vegetative phase of the crop [4,5,6,7]. Like many sap-sucking insects, aphids cause damage in susceptible cowpea cultivars directly by altering plant metabolism and feeding on plant nutrients and indirectly by transmitting plant-pathogenic viruses, especially the aphid-borne cowpea mosaic viruses [6,8]. Cowpea aphids are known to release a honeydew substance that blocks plant respiration and stimulates the development of a black mold which curtails photosynthesis [9]. The adult insect produces eggs internally, giving live birth to nymphs that quickly mature into reproductive adults within 2 to 3 days [10]. It has been noted that aphids can wipe out the entire cowpea crop when the attack coincides with drought occurrence at seedling stage [2]. Aphid control measures on cowpea include the use of insecticides and other cultural, physical, and biological methods [11]. These control approaches are inefficient and not environmentally friendly or cost effective [11,12]. The use of resistant cultivars is the best control method which can also easily be combined with other methods to achieve holistic integrated pest management (IPM) strategies [12]. Consequently, there has been increased effort in the search for sources of resistance to cowpea aphids and better understanding of the nature and mechanism of resistance. Several sources of resistance to aphids have been identified in different breeding programs [5,13,14,15,16]. Aphid resistance is a priority trait in the cowpea breeding program of the International Institute of Tropical Agriculture (IITA). Some improved resistant lines have been developed and distributed to farmers in many countries [9,10,11,12,17,18,19]. The identified resistant sources have been used in many studies to elucidate the mechanism of resistance to *A. craccivora*. These studies revealed slow growth and multiplication rates of aphids and, therefore, lower damage to resistant lines, indicative of strong antibiosis [6,20,21]. However, disruption of aphid stylet penetration activities followed by a non-preference of the host plant (antixenosis) or a combination of both antibiosis and antixenosis mechanisms have also been reported [4,6]. Further investigations have implicated tolerance as a third mechanism, which is exhibited by plants surviving beyond 21 days under high aphid infestation [12]. These observations, coupled with studies using model plant *Arabidopsis,* have reinforced our understanding of the basal defense mechanisms against aphid feeding [22], although the molecular basis of the gene mediated aphid resistance remains elusive. Some insights into resistance gene-mediated defense to aphids have been shown in tomato [23], soybean [24] and the model legume *Medicago truncatula* Gaertn [25], with evidence that specific defense signaling pathways are elicited in resistant but not susceptible plants.

Genetic studies postulated single, dominant genes as the main control of resistance to aphids in cowpea [5,15,26]. This has been backed by allelism tests which indicated that some of the identified sources of resistance displayed independent genes, each segregating in a dominant model [10,14]. Reported quantitative trait loci (QTL) for aphid resistance in cowpea have supported the notion of single, dominant gene actions. For instance, in an F_2_ population involving a resistant cultivar IT84S-2246-4, a restriction fragment length polymorphism (RFLP) marker linked to a major effect resistance to cowpea aphids was identified on linkage group 1 [27]. Additionally, in a bi-parental population involving a resistant line IT97K-556-6, [16] identified one major and one minor aphid resistance QTLs on chromosomes Vu02 and Vu05, respectively, and the major QTL segregated in a 3:1 ratio model in a related F_2_ population. Single-nucleotide polymorphism (SNP) markers flanking these two QTLs have been deployed in marker-assisted breeding for aphid resistance [5,18]. Recently, a genome-wide association study (GWAS) conducted with a low-density marker system (1047 SNPs) on 338 cowpea accessions obtained from USDA-GRIN germplasm database highlighted some association signals for aphid resistance on two contigs of the cowpea genome [28]. A detailed genome-wide scan with high density markers would provide a clearer picture of the aphid resistance landscape in cowpea, opening doors for the development of durable resistance marker systems.

Recently, existing novel cowpea genetic and genomic resources are making it possible to discover useful molecular markers for deployment in breeding [2]. Among the resources so far developed, [29] designed an Illumina iSelect Consortium Array consisting of 55,496 SNPs, creating an excellent and highly dense SNP genotyping platform for cowpea. In addition, IITA constituted 365 cowpea accessions from a collection of over 15,000 world cowpeas to form a sub-set panel commonly referred to as the mini-core population [2,30]. These resources offer a great opportunity for QTL discovery and marker development for use in breeding for aphid resistance and other key traits desired in cowpea. The quest to locate new alleles for aphid resistance in cowpea is backed by the observations that previously identified major resistance genes have broken down and are no longer effective [2,12,16]. In addition, multiple ecotypes of *A. craccivora* have been reported [5,11,14,31,32], adding complexity to the management of this insect pest. Dependence on single resistance genes is therefore not helpful since it can often be quickly circumvented by the pest. Efforts toward validation of previously mapped alleles and discovery of loci involved in aphid resistance are needed as these would aid the pyramiding of genes for durable resistance to this pest. The present study exploited the diversity in the IITA cowpea mini-core collection coupled with high-density SNP marker system to expose new loci involved in resistance to *A. craccivora* across the entire cowpea genome. The study used phenotyping data from the diverse accessions grown under artificial aphid infestation in screening cages at IITA, Kano station (Nigeria). Resistance to *A. craccivora* was assessed based on severity of damage on cowpea leaves. High density SNPs were deployed in GWAS followed by blast search for candidate gene functions. The study contributes to the efforts of establishing a marker-aided selection platform for aphid resistance in cowpea and eventual development of cultivars that are resistant to aphids.

## 2. Materials and Methods

### 2.1. Plant Materials

The present study used a set of 365 mini-core accessions, constituted from a world collection maintained by IITA [2,30]. The mini-core accessions have been genotyped with high density SNPs and were carefully sampled to represent the diversity in global cowpea collections maintained at IITA gene bank [2,30]. These were exploited in the present study in order to understand the link between phenotypic and DNA variations for aphid resistance in cowpea. The IITA mini-core population contains diverse collections of cowpea landraces originating from more than 50 different countries across the world [30,33,34,35].

### 2.2. Aphid Culturing

Aphid ecotype from Kano, Nigeria was used in this study. The aphids were collected from cowpea fields at the IITA Minjibir Research Farm, Latitude 12°14′35.30″ N and Longitude 8°66′62.10″ E located at about 45 km from Kano City (Kano State, Nigeria) [7]. Aphids were multiplied on susceptible cowpea variety TVx3236 to raise sufficient inoculum. Details of aphid culturing procedure has been described by [36].

### 2.3. Experimental Layout

Three sets of the same experiment were established over time to phenotype the mini-core accessions for reaction to cowpea aphid infestation. The accessions were evaluated in screening cages at the IITA, Kano station, Nigeria. The accessions which included resistant and susceptible checks, namely TVu-801 and TVx3236 were planted in wooden trays measuring 1 m width × 1 m length, 0.11 m height, filled with topsoil and covered with insect-proof nets (Supplementary Figure S1). Each experimental set was laid out in an augmented design where each wooden tray was considered as a block, planted with 12 entries (that is 10 mini-core accessions and 2 checks). The resistant and susceptible checks were repeated in each block, creating 36 pseudo-replications. Ten seeds per accession were planted in a row at a spacing of 8 cm within the row and 8 cm between rows.

### 2.4. Aphid Infestation and Damage Severity Score

**Figure 1:**
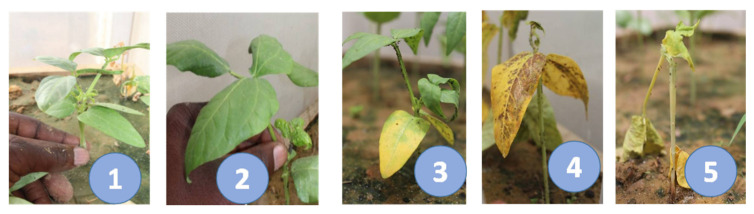
Aphid damage severity rating scale. The damage scored on a scale of 1–5 [7]), where 1 = no damage or symptoms on the leaves, 2 = fewer symptoms (one or two leaves showing aphid feeding symptoms), 3 = seedling leaves are partially yellow, 4 = seedling leaves totally yellow, and 5 = seedling plants are dead. Photo credit: Abou Togola.

Artificial aphid infestations were accomplished using the aphid ecotype from Kano, Nigeria. Responses of accessions to aphid infestation in all experiments were rated based on aphid damage severity at 21 days after infestation as described by [36]. Aphid damage severity was scored on a scale of 1–5, where 1 = no damage or symptoms on the leaves, 2 = fewer symptoms (one or two leaves showing aphid feeding symptoms), 3 = seedling leaves are partially yellow, 4 = seedling leaves totally yellow, and 5 = seedling plants are dead (Figure 1).

### 2.5. Phenotypic Data Analysis

Adjusted mean values of phenotypic data collected from cowpea accessions were obtained by conducting augmented analysis of variance using ‘*augmentedRCBD*’ package in R [37,38]. The model accounts for the effects of the block, test entry and check entry. Treating the check entry as fixed while the block and the test entry random, the augmented model estimates variance components based on the following expected mean square expressions [37,38]:

$$\mathrm{Residual}:\;EMS=\sigma^{2}\varepsilon$$

$$\mathrm{Test}\;\mathrm{entry}:\;MS\tau =\sigma^{2}\varepsilon +\sigma^{2}\tau$$

$$\mathrm{Check}\;\mathrm{entry}:\;MS_{k}=\sigma^{2}\varepsilon +\sum_{1}^{k}\mu^{2}k-\frac{1}{k}{\left(\sum_{1}^{k}\mu k\right)}^{2}$$

$$\mathrm{Block}:\;MS\beta =\sigma^{2}\varepsilon +k\sigma^{2}\beta$$

where $\sigma^{2}\varepsilon$ is the error variance, $\sigma^{2}\tau$ is the test entry variance, $\sigma^{2}\beta$ is the block variance, μk is the mean of the checks and *k* is the number of check entries used. The variance components were utilized to estimate several genetic variability statistics for aphid damage severity including phenotypic variation, genotypic variation, broad sense heritability, expected genetic advance among other measures. The analysis also generated frequency distributions that were used to describe the phenotypic distribution of the accessions based on aphid damage severity on cowpea leaves.

### 2.6. SNP Genotype Data Acquisition

The IITA mini-core accessions used in the present study were genotyped using the Cowpea iSelect Consortium Array containing 51,128 SNPs [35,39]. Genotyping was conducted at the University of Southern California Molecular Genomics Core facility (Los Angeles, CA, USA). SNPs were called using GenomeStudio software V.2011.1 (Illumina, Inc., San Diego, CA, USA) after alignment to the IT97K-499-35 reference genome developed and assembled by [1]. SNP data were filtered for downstream analyses using TASSEL for windows version 5.2.50 [40], allowing 20% missing data and minor allele frequency (MAF) less than 5%, leaving a total of 40,405 SNPs for downstream analyses. After filtering the SNP data for quality control, the distributions of 40,405 SNPs on the 11 chromosomes of cowpea were examined by generating an SNP density plot using memory-efficient, visualization-enhanced, and parallel-accelerated R package “*rMVP*” [41], with a window size of 1 Mb.

### 2.7. Genome-Wide Association Analysis

Genome-wide association study was implemented in *rMVP* package [41]. Four different models were tested: (i) general linear model (GLM) accounting for population structure (Q) [42]; (ii) mixed linear model (MLM) accounting for population structure (Q) and kinship (K) [43]; and (iii) fixed and random models circulating probability unification (FarmCPU), accounting for population structure (Q) and kinship (K) [44], all accomplished with *rMVP* package. Each model tested the same hypothess—Ho: There is no association between SNP and trait; Ha: There is an association between SNP and trait. For verification purpose, similar models with exception of FarmCPU were also fitted using TASSEL version 5.2.50. To authenticate the association signals, the accessions were sorted by the two alleles at each peak SNP and boxplots were generated using the R program to examine the difference in traits mean and dispersion within the allelic groups. A traditional two-tailed *t*-test (at alpha *α* = 0.05) was conducted for each peak SNP to verify if the two alleles of an SNP significantly differentiated between the group’s traits’ means of the accessions.

The GWAS significance threshold used in this study was determined by correcting for multiple testing through control of false discovery rate (FDR) [45]. The FDR approach aims at controlling the proportion of significant results that are in fact type I errors, and it has been praised to be more powerful in controlling the proportion of falsely rejected hypothesis than the conservative Bonferroni procedure [45,46,47]. We implemented FDR in R environment using the *p.adjust*() function, with the method set to “fdr”, which adjusts the GWAS *p*-values based on Benjamini and Hochberg (“BH”) procedure [45]. An average FDR threshold was then computed from the adjusted *p*-values at 5% probability level as follows:

$$FDR=\left(\alpha \times 100\right)/\left(\sum_{i}^{1}p.adjust\right)$$

where FDR is the false discovery rate threshold, α is the acceptable level of type I error which was set at 0.05 in the present study, $\overset{1}{\underset{i}{\sum }}p.adjust$ is the sum of adjusted *p*-values for each SNP extracted from the R output; that is, $\overset{1}{\underset{i}{\sum }}p.adjust$ = 39,880.05 in our case. The −log10^(*FDR*)^ was then taken to establish the significant threshold for the GWAS results. Consequently, the *FDR* threshold in the present study was computed as:

*FDR* = [(0.05 × 100/39,880.05) = 1.25 × 10^−4^; hence, −log10^(*FDR*)^ = 3.9

### 2.8. Candidate Gene Prediction

To explore the likely genes responsible for the detected association signals, the positions of peak SNPs were searched along the annotated genome (v1.1) of elite IITA cowpea variety IT97K-499-35 [1] using the genome browser (JBrowse) in Phytozome 13 (https://phytozome-next.jgi.doe.gov/info/Vunguiculata_v1_1, accessed on 15 September 2022). Predicted genes within the peak SNP regions were further explored for their annotated biological functions in relation to homologs in other crops, especially common bean (*Phaseolus vulgaris*), soybean (*Glycine max*), barrelclover (*M. truncatula*) and *Arabidopsis thaliana*, via both Phytozome and the *V. unguiculata* Gene expression atlas developed by [48].

## 3. Results

### 3.1. Phenotypic Assessments

**Figure 2:**
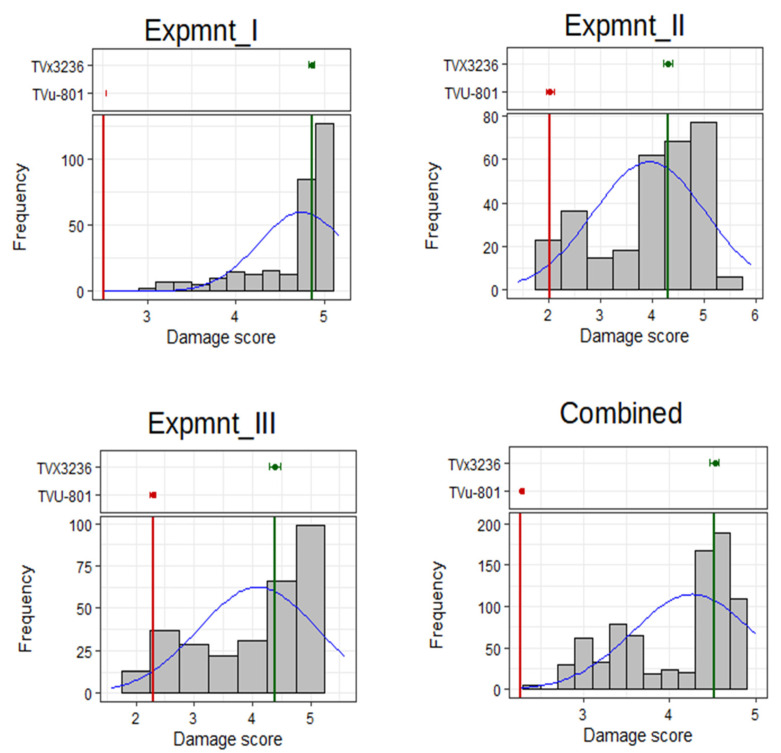
Phenotypic distributions for aphid damage severity in the cowpea mini-core population that were evaluated in three screenhouse experiments. The positions of the resistant check TVu-801 and the susceptible check TVx3236 are shown by the red and green vertical lines, respectively.

Cowpea mini-core accessions exhibited significant variability (*p* < 0.001) in aphid damage severity rated 21 days after infestation across all the three experiments conducted (Table 1). The observed differences among accessions were displayed as a range of phenotypes from resistant plants with minimal aphid damage to complete susceptibility indicated by severe damage symptoms on cowpea leaves. These differences were supported by the observed significant difference (*p* < 0.001) between the resistant and susceptible checks that were included in the study (Table 1). The extent of phenotypic variation for aphid resistance among accessions based on damage severity is displayed by the frequency distributions (Figure 2). The distributions were fairly skewed, with the left tail carrying resistant accessions that were identifiable by the positions of the resistant and susceptible checks. Aphid damage severity was seemingly higher in experiment I compared to experiments II and III. This is reflected in the left tails of frequency distributions where no accession was better than the resistant check in experiment I, while the reverse was true in experiments II and III.

Dissection of the observed variation into its component sources revealed that genetic variances among accessions based on aphid damage severity were higher than the environmental variances in all the three experiments (Table 2). Similar statistical patterns were obtained from combined experimental data. Consequently, moderate to high estimates for genotypic coefficient of variation (GCV), broad sense heritability (H^2^_BS_) and genetic advance (GA) based on aphid damage severity were recorded across the experiments.

### 3.2. SNP Density and Linkage Disequilibrium

**Figure 3:**
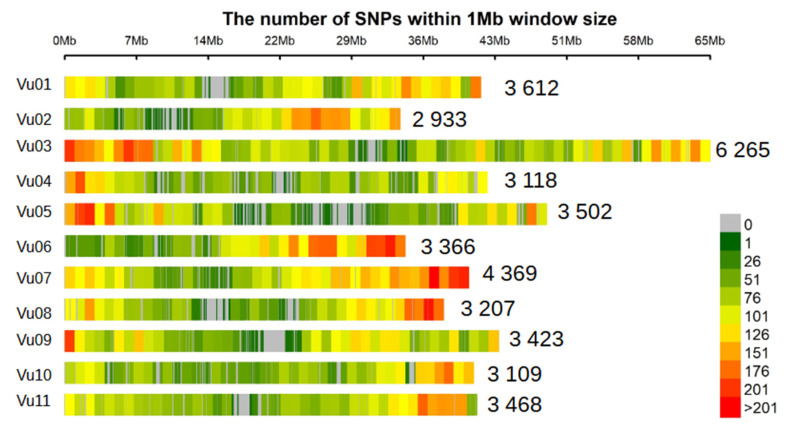
SNP distributions on 11 chromosomes of cowpea. A heat map is presented showing chromosomal regions with high number of SNP within 1 Mb window size. The horizontal axis displays the chromosome length. Legend (0–201) insert indicates the SNP density; on top of each chromosome there is an insert reflecting the total number of SNPs per chromosome.

The distribution of 40,405 polymorphic SNP markers on the 11 chromosomes of cowpea is graphically presented in Figure 3, and the SNP genotype data is available in Supplementary Materials Table S1. The SNPs were spread over the 640.6 Mb of the cowpea genome, with each SNP occurring at an approximate interval of 0.016 Mb. The total number of SNPs per chromosome ranged from 2933 on Chromosome Vu02 to 6265 on Chromosome Vu03. The high-density of SNPs in this panel offers opportunity for high resolution QTL mapping. A genome-wide linkage disequilibrium was examined by calculating pairwise squared correlations (r^2^) between SNPs. LD in this cowpea population was found to decay to r^2^ = 0.2 at a physical distance of ~200 kb (Supplementary Materials Figure S2). This value was considered as the average extent of LD for this cowpea collection. The relatively fast LD decay observed in this panel is indicative of its advantageous potential for reducing QTL intervals and fine mapping of candidate genes for aphid resistance.

### 3.3. Genome-Wide Association Signals

**Figure 4:**
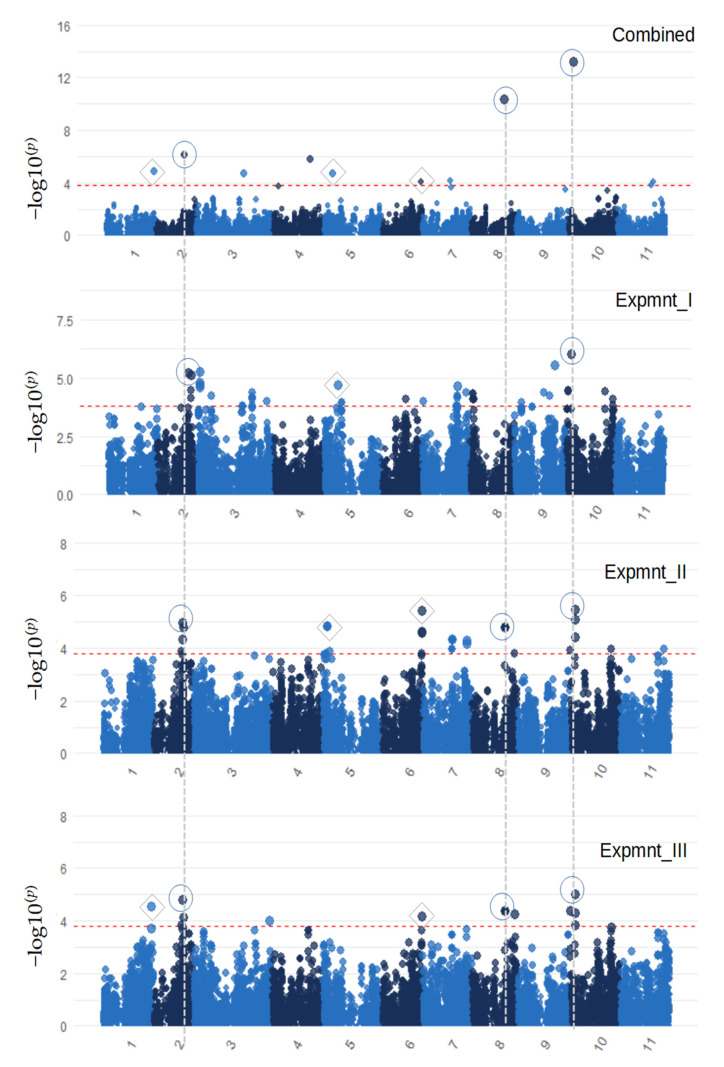
Manhattan plots depicting genome-wide association signals for resistance to cowpea aphid ecotype collected from Kano, Nigeria. The signals are based on SNP association with aphid damage severity data collected from three experiments. GWAS results are presented for combined analysis using data across three experiments, and for individual experiments represented here as Epmnt_1, Expmnt_II and Expmnt_III. The vertical dash lines with circles highlight the major regions that were mapped in more than one experiment. Horizontal dash lines indicate GWAS significance threshold determined by FDR. Minor peaks are highlighted by diamond symbols. *p*-values expressed as −log10^(^*^p^*^)^ and chromosome names are shown on Y and X axes, respectively.

A genome-wide scan based on adjusted means for aphid damage severity depicted three major association signals on chromosomes Vu02, Vu08 and Vu10 and two minor ones on chromosomes Vu01 and Vu06 (Figure 4). These five signals were consistently identified using at least two individual data sets in addition to the combined data. There were, however, some peaks on chromosomes Vu03, Vu04, Vu05 and Vu07 that were significant but consistent. When a more conservative Bonferroni GWAS threshold was considered, the three major signals on Vu02, Vu08 and Vu10 persisted while the others that had met the FDR threshold became insignificant (Supplementary Materials Figure S3). The three major effect regions on chromosomes Vu02, Vu08 and Vu10 that displayed highly significant associations for aphid resistance were represented by peak SNPs 2_24304 [−log^(p)^ = 6.19], 2_22525 [−log^(p)^ = 10.37] and 2_43876 [−log^(p)^ = 13.19, respectively (Table 3, Supplementary Materials Table S2). Overall, a total of 20 SNPs were found to contribute significantly to aphid resistance variation in the cowpea mini-core population. These SNPs are being validated in bi-parental populations to ascertain their consistencies in different genetic backgrounds.

**Figure 5:**
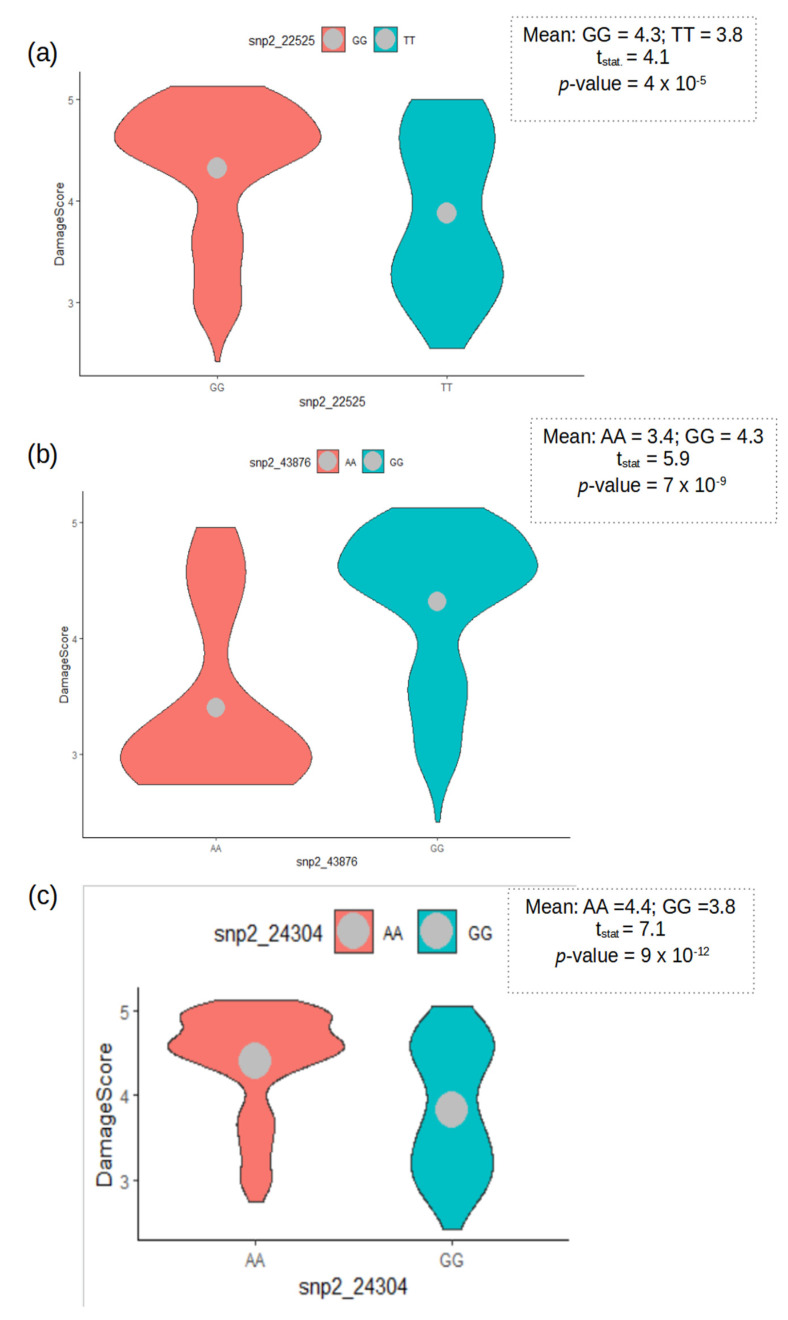
Single-marker differentiation of accessions by the two alleles of each peak SNP at the three major association signal regions on: (**a**) chromosome Vu08 represented by SNP marker 2_22525; (**b**) chromosome Vu10 represented by SNP marker 2_43876; (**c**) chromosome Vu02 represented by SNP marker 2_24304. Boxplots depict extent of dispersion within allelic categories of peak SNPs. Square dash-line box inserts show the results of *t*-tests for differences between the means of two groups categorized based on SNP marker alleles.

To assess how the alleles at the candidate loci influenced variations among accessions for resistance to aphids, we categorized the accessions based on the two alleles of each peak SNP of the three major regions and tested the differences in phenotype means between the allelic groups. Single-marker analysis based on student *t*-test revealed highly significant (*p* < 0.001) differences between the allelic group means for the three peak SNPs (Figure 5). Alleles responsible for resistance to aphid at the mapped regions had lower mean aphid damage severity relative to the alternative alleles. Overall, the accessions carrying favorable alleles at each peak SNP were identified as being more resistant than those carrying the alternative alleles. The significant differentiation between accessions based on alleles at each candidate locus supported the significant associations signals detected by GWAS.

### 3.4. Gene Predictions and Functions

A summary of the proximal genes to the peak SNPs positions and the annotated gene functions are presented in Table 4. A Phytozome gene search identified five genes that were proximal to the position of representative peak SNPs. The genes uncovered within the three major regions that were associated with resistance to aphid on chromosomes Vu10, Vu08 and Vu02 included *Vigun10g031100.1* (Leucine-Rich Repeat-containing Protein), *Vigun08g030200.1* (solute carrier family 45) and *Vigun02g088900.1* (Cysteine-rich TM module stress tolerance (CYSTM)), respectively. SNP Variants 2_43876 and 2_22525 were located at 2639 bp and 346 bp respectively upstream from the start position of the associated genes on chromosomes Vu10 and Vu08, while variant 2_24304 on chromosome Vu02 was positioned at 3123 bp upstream. The other minor regions on chromosomes Vu01 and Vu06 harbored *Vigun01g233100.1* and *Vigun06g224900.1* genes, respectively. The SNP variant 2_28582 on chromosome (Vu01) was situated at 2184 bp downstream of gene, while 2_30711 on chromosome Vu06 was 107 bp upstream from the start position of the gene in question. A search for homology via Phytozome and BLAST hits from the *V. unguiculata* gene expression atlas (VuGEA) revealed that these candidate genes had homologs in common bean (*P. vulgaris*), soybean (*G. max*), barrelclover (*M. truncatula*) and *A. thaliana.*

## 4. Discussion

*A. craccivora* is a widespread insect pest of cowpea in Africa and other parts of the world. The devastating impact of this insect on cowpea has over time attracted research attention towards understanding the mechanisms of resistance to the pest and effective control strategies. Deployment of molecular markers has been emphasized as the best approach to enhance breeding for resistance to this economically important insect pest [5,10,16,18,28]. The success of the molecular breeding approach, however, requires explorations and discovery of markers closely tagging the resistance loci. The present study has added to the body of knowledge by uncovering new genomic regions involved in resistance to aphids in cowpea. The study achieved this objective by mining the diversity in 365 cowpea mini-core accessions maintained by the IITA. The 365 accessions used in the present study came from more than 50 countries, the rich diversity and structure of which were previously described based on GBS data [30,34]. This mini-core panel is, therefore, suitable for exploration of genetic control of key traits in cowpea given the broad diversity it encompasses. The reactions of the cowpea accessions to artificial infestation using *A. craccivora* from Kano, Nigeria showed considerable ranges in aphid damage severity. Frequency distributions of accessions based on aphid damage severity were skewed, which is suggestive of few major QTLs or genes driving the observed pattern [49,50]. Consequently, the genotypic differences among accessions in the present study were discernable, allowing extraction of aphid-resistant accessions and a feasible downstream investigation of underlying genetics. Previous field-based studies exhibited clear differences among cowpea lines in their responses to aphid infestations, and these allowed identification of some resistance sources [5,7,10,12,13,16,51,52]. Genetic studies on aphid resistance have put forward a monogenic inheritance theory [14,15,26]; however, resistance sources such as TVu-3000, which has been popularly used as a parent in breeding for aphid resistance, have become ineffective due to resistance breakdown [2,51]. In addition, it has been reported that there is considerable variability within the cowpea aphid species resulting in existence of different ecotypes or biotypes [11,14,32,53]. These observations, coupled with the fact that resistance genes from different sources are non-allelic and independent [5,15], suggest the need to discover the key genes involved in aphid resistance and pyramid them in the same background for a robust and durable resistance.

The scanning of the entire cowpea genome in the present study, made possible by a highly dense SNP marker system, in addition to high genetic diversity among the accessions, allowed us to uncover five loci underlying resistance to cowpea aphid ecotype from Kano, Nigeria. Some of the loci identified in the present study were proximal to the chromosomal regions previously mapped [5,16]. The first genetic linkage mapping for aphid resistance in cowpea used restriction fragment length polymorphism (RLFP) marker technology and identified a marker, bg4D9b, linked to the aphid resistance gene (*Rac1*) in cowpea line IT84S-2246-4 on linkage group 1 [27]. However, this single dominant gene has since become ineffective against aphids [2]. Given the recent developments in cowpea genomics, 1536 EST-derived SNPs were utilized in linkage analysis to identify two QTLs for aphid resistance, *QAc-vu1.1* and *QAc-vu7.1,* on chromosomes Vu05 and Vu02, respectively, using 92 recombinant inbred lines (RILs) from a cross between susceptible CB27 line from UCR and resistant IT97K-556-6 line from the IITA [16]. HarvEST BLAST search of the source sequences of flanking SNPs from [16] against the cowpea reference genome sequence (IT97K-499-35 v1.0) at http://harvest-web.org/hweb/mainmenu.wc (accessed on 15 September 2022), mapped the two QTLs *QAc-vu1.1* and *QAc-vu7.1* on chromosomes Vu05 (37.483 Mb) and Vu02 (25.345 Mb), respectively. In the present study, a significant association for resistance to the Kano, Nigeria aphid ecotype was detected on chromosome Vu02, flagged by SNP marker 2_24304 at position 24.352 Mb; hence, it was just 0.99 Mb away from *QAc-vu7.1*. Recently, using 169 F_2_ plants from a cross of Apagbaala’ (Local susceptible) × SARC 1-57-2 (Resistant) and validation in the CB27 × IT97K-556-6 population, a novel locus for aphid resistance tagged with codominant SSR marker *CP 171F/172R* was identified [5]. The physical position of the SSR marker was mapped at 30.514 Mb on Vu10 of the cowpea genome. This locus was independent of other mapped loci, and the authors asserted that the aphid resistant sources SARC 1-57-2 and IT97K-556-6 carry different resistance genes. The present study, based on a genome-wide scan with high density SNPs, identified a distant association on the same chromosome (Vu10) at position 4.094 Mb, which is 26.42 Mb away from the *CP 171F/172R* locus, suggesting that this is a novel locus. Additionally, ref. [28] reported detection of some two aphid resistance loci on the contigs of the ‘Assembled Cowpea WGS Sequences v0.03′ [39], using 338 cowpea accessions, genotyped with 1047 SNPs from genotyping by sequencing (GBS) platform. The loci were tagged by SNP markers C35011941_894 and Scaffold30061_3363 [28]. A BLAST search of these scaffolds/contigs sequences through HarvEST server placed both loci on chromosome Vu03 at positions 56.942 Mb (*C35011941_894*) and 16.443 Mb (*Scaffold30061_3363*). Although not consistent, our study detected a locus Vu03 flagged by SNP variant 2_06664 at position 40.68 Mb, approximately 16 Mb away from the *C35011941* locus reported [28]. Moreover, the present study identified other loci on chromosomes Vu08 (Pos 27.78 Mb), Vu06 (Pos 33.32 Mb) and Vu01 (Pos 40.5 Mb) that were independent of previously reported QTLs and are therefore novel.

We also explored the candidate genes in the vicinity of regions that were detected by GWAS to be contributing to aphid resistance. The study exploited the publicly available cowpea genomic resources such as the whole-genome shotgun (WGS) assembly [39], available through HarvEST:Web (http://harvest-web.org/, accessed on 15 September 2022); the genome assembly of cowpea IT97K-499-35 [1], accessible through Phytozome (phytozome.jgi.doe.gov, accessed on 15 September 2022); HarvEST BLAST Server [54], also accessible via HarvEST:Web; and cowpea gene expression atlas [48], to elucidate the candidate genes. Five genes were identified at the mapped regions that were associated with aphid resistance. The functional annotations of these genes were consistent with their homologs from Arabidopsis, common bean, soybean and medicago. Different studies have implicated expression of these genes under array of conditions including salinity [55], mechanical wounding, insect feeding [56], pathogens and stress signaling [57] and resistance to insects in different plants [58,59,60]. The evidence presented here regarding the identified genes suggests their significant roles in plant defense systems and particularly resistance to insects. Validation studies to confirm the mapped loci and gene expression analysis under aphid infestation would enhance confidence in deploying these loci in marker-assisted breeding for aphid resistance in cowpea. The 20 significant SNPs identified in this study are being validated in different genetic backgrounds for potential deployment in marker-aided breeding for aphid resistance in cowpea.

## 5. Conclusions

Our study revealed sufficient genetic variation for aphid resistance in the IITA mini-core population. Variation for aphid resistance in this population was found to be under the control of three major genomic regions on chromosomes Vu10, Vu08 and Vu02 in addition to two other minor regions. The mapped regions harbored several genes, including *Vigun01g233100.1*, *Vigun02g088900.1*, *Vigun06g224900.1*, *Vigun08g030200.1* and *Vigun10g031100.1,* which were proximal to the peak regions, the functional annotations of which relate to plant defense system. Our study uncovered new loci for aphid resistance thereby contributing towards a better understanding of the genetic control of this insect pest in cowpea. The SNP markers that were associated with aphid resistance are being tested in our program for consistent associations in different genetic backgrounds. Once validated, these SNP markers will be deployed in marker-aided breeding programs for accelerated development of aphid resistant lines.

## Figures and Tables

**Table 1:** Analysis of variance for aphid damage severity in the cowpea mini-core population evaluated in three experiments, each established in an augmented block design.

Aphid Damage Severity Experiment I				
**Source**	**DF**	**SS**	**MS**	**F-value**
Treatment	358	250.46	0.70	17.90 ***
Check entry	1	101.72	101.72	2602.31 ***
Test entry	356	78.24	0.22	5.62 ***
Test vs. Check	1	70.50	70.50	1803.66 ***
Block	35	0.91	0.03	0.66 ^ns^
Residuals	37	1.45	0.04	
**Aphid Damage Severity Experiment II**				
**Source**	**DF**	**SS**	**MS**	**F-value**
Treatment	319	475.60	1.49	5.91 ***
Check entry	1	91.40	91.43	362.67 ***
Test entry	317	349.00	1.10	4.37 ***
Test vs. Check	1	35.10	35.14	139.40 ***
Block	34	6.90	0.20	0.81 ^ns^
Residuals	34	8.60	0.25	
**Aphid Damage Severity Experiment III**				
**Source**	**DF**	**SS**	**MS**	**F-value**
Treatment	319	420.20	1.32	5.50 ***
Check entry	1	76.80	76.76	320.62 ***
Test entry	317	310.70	0.98	4.09 ***
Test vs. Check	1	32.80	32.77	136.89 ***
Block	34	5.00	0.15	0.61 ^ns^
Residuals	34	8.10	0.24	
**Aphid Damage Severity Combined**				
**Source**	**DF**	**SS**	**MS**	**F-value**
Treatment	366	906.60	2.48	7.16 ***
Check entry	1	269.30	269.29	778.07 ***
Test entry	364	492.10	1.35	3.90 ***
Test vs. Check	1	145.20	145.20	419.45 ***
Experiment	2	116.60	58.28	168.39 ***
Residuals	838	290.00	0.35	

ns, *p* > 0.05; ***, *p* <= 0.001; DF, degree of freedom; SS, sums of squares; MS, mean square; F-value, F-statistical test value.

**Table 2:** Genetic variability statistics for aphid damage severity in the cowpea mini-core population evaluated in three screenhouse experiments.

Statistics *	Experiment I	Experiment II	Experiment III	Combined
Mean	4.75	3.95	4.10	4.27
Min	2.51	1.42	1.42	2.27
Max	5.17	5.92	5.92	5.00
SE	0.03	0.06	0.06	0.02
CD_(α=0.05)_	0.09	0.24	0.24	0.98
CV (%)	4.32	13.17	12.36	14.23
GCV (%)	8.94	23.31	21.01	23.49
PCV (%)	9.86	26.55	24.17	27.23
H^2^_BS_ (%)	82.22	77.10	75.57	74.40
GA	0.80	1.67	1.54	1.78
GAM (%)	16.73	42.23	37.68	41.80

Abbreviations: CV = coefficient of variation; SE = standard error of mean; CD = critical difference between two means at 5% α level computed as [√(2EMS/b)] * (t_0.05,DFerror_), where b is the number of blocks and EMS is the error mean square; GCV = genotypic coefficient of variation; PCV = phenotypic coefficient of variation; H^2^_BS_ = broad sense heritability; GA = genetic advance; GAM = genetic advance as percent of mean.

**Table 3:** Representative significant SNPs that were associated with resistance to *A. craccivora* rated based on aphid damage severity in the cowpea mini-core population.

Experiment	SNP Name	Chromosome	Position(bp)	Allele	−Log10^(^*^p^*^)^	R^2^
	2_43876 ^e^	Vu10	4,096,878	G/A	13.19	8.02
	2_22525 ^d^	Vu08	27,780,564	G/T	10.37	4.34
Combined	2_24304 ^b^	Vu02	24,348,915	A/G	6.19	6.06
	2_28582 ^a^	Vu01	40,503,572	G/A	4.90	3.84
	2_30711 ^c^	Vu06	33,320,998	G/A	4.10	5.43
Experiment I	2_43876 ^e^	Vu10	4,096,878	G/A	6.02	5.49
	2_16937	Vu02	28,397,818	C/T	5.13	4.00
	2_37658	Vu02	26,139,374	T/C	5.21	5.00
	2_30970	Vu02	28,402,526	G/A	4.47	3.70
Experiment II	2_43876 ^e^	Vu10	4,096,878	G/A	5.09	5.40
	2_03743 ^g^	Vu10	4,104,605	T/C	5.47	5.70
	2_13144	Vu10	4,058,697	G/A	4.43	4.10
	2_38695 ^f^	Vu08	27,786,635	T/C	4.80	4.30
	2_22525 ^d^	Vu08	27,780,564	G/T	4.80	4.34
	2_22524	Vu08	27,782,280	T/G	4.80	4.30
	2_24304 ^b^	Vu02	24,348,915	A/G	4.96	5.54
	2_24860	Vu02	25,636,527	T/C	4.82	3.60
	2_47978	Vu02	24,342,315	A/T	4.35	5.00
	2_30711 ^c^	Vu06	33,320,998	G/A	5.41	5.43
	2_34496	Vu06	33,320,879	T/A	4.60	4.30
	2_54768	Vu06	33,347,257	C/T	4.62	4.70
Experiment III	2_43876 ^e^	Vu10	4,096,878	G/A	4.32	4.60
	2_03743 ^g^	Vu10	4,104,605	T/C	5.01	5.40
	2_28582 ^a^	Vu01	40,503,572	G/A	4.56	4.47
	2_22525 ^d^	Vu08	27,780,564	G/T	4.39	4.21
	2_38695 ^f^	Vu08	27,786,635	T/C	4.39	4.20
	2_22524	Vu08	27,782,280	T/G	4.39	4.20
	2_55335	Vu08	35,392,403	C/T	4.27	3.40
	2_24304 ^b^	Vu02	24,348,915	A/G	4.82	5.76
	2_30711 ^c^	Vu06	33,320,998	G/A	4.17	4.33

SNPs labeled with the same letters a, b, c, d, e, f, g were significant in more than one instance of experimental analysis and were therefore consistent across experiments. R^2^ value expressed as percentage of phenotypic variance explained; −Log10(*p*) is a measure of significance level alternative to LOD (logarithm of odds) score.

**Table 4:** Predicted genes proximal to the position of representative SNPs that were associated with resistance to *A. craccivora* rated based on aphid damage severity in the cowpea mini-core population.

SNP	Chr ^a^	Pos(Bp) ^b^	Gene	GPos(Bp) ^c^	GDist (Bp) ^d^	Gene Functional Annotation
2_43876	Vu10	4,096,878	*Vigun10g031100.1*	4,094,239	−2639	Leucine-Rich Repeat-containing Protein
2_22525	Vu08	27,780,564	*Vigun08g030200.1*	27,780,218	−346	Solute carrier family 45
2_24304	Vu02	24,348,915	*Vigun02g088900.1*	24,352,038	3123	Cysteine-rich TM module stress tolerance (CYSTM)
2_28582	Vu01	40,503,572	*Vigun01g233100.1*	40,501,388	−2184	PROTEIN NRT1/PTR FAMILY 5.1
2_30711	Vu06	33,320,998	*Vigun06g224900.1*	33,321,105	107	DNAJ HOMOLOG SUBFAMILY C MEMBER

^a^ Chromosome, ^b^ SNP position in base pairs; ^c^ gene position measured in base pairs; ^d^ distance in base pairs from start position of the gene to the position of peak SNP. The negative [−] sign indicates that start position of the gene is earlier than that of the peak SNP marker.

## Data Availability

All data reported in this study have been provided as Supplementary Materials.

## Supplementary Materials

The following supporting information can be downloaded at: https://www.mdpi.com/article/10.3390/genes13112002/s1, Figure S1: Experimental layout for aphid screening showing a setup of the wooden trays each measuring 1 m × 1 m and complete insect-proof cages planted with cowpea mini-core accessions and inoculated with *A. craccivora*; Figure S2: Genome-wide linkage disequilibrium (LD) decay showing r^2^ values against physical distance (kb) for cowpea mini-core accessions; Figure S3: Manhattan and QQ plot depicting three major genomic regions for resistance to cowpea aphid on chromosomes Vu10, Vu08 and Vu02 after considering a more conservative Bonferroni significant threshold; Table S1: SNP genotype data for the 365 IITA cowpea mini-core accessions; Table S2: FarmCPU GWAS statistics used to generate Manhattan plots.
