# Supplementary figures and images for: A Genome-Wide Scan Divulges Key Loci Involved in Resistance to Aphids (Aphis craccivora) in Cowpea (Vigna unguiculata)
Source: Genes (Basel). 2022 Nov 1;13(11):2002. doi: 10.3390/genes13112002 (PMC9690070; doi:10.3390/genes13112002)

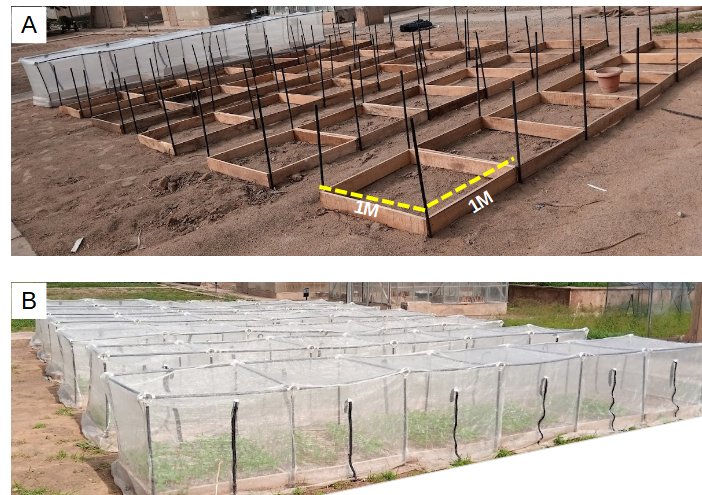

Supplement: Supplementary file 1 [file genes-13-02002-s001.zip › Figure S1.tif]

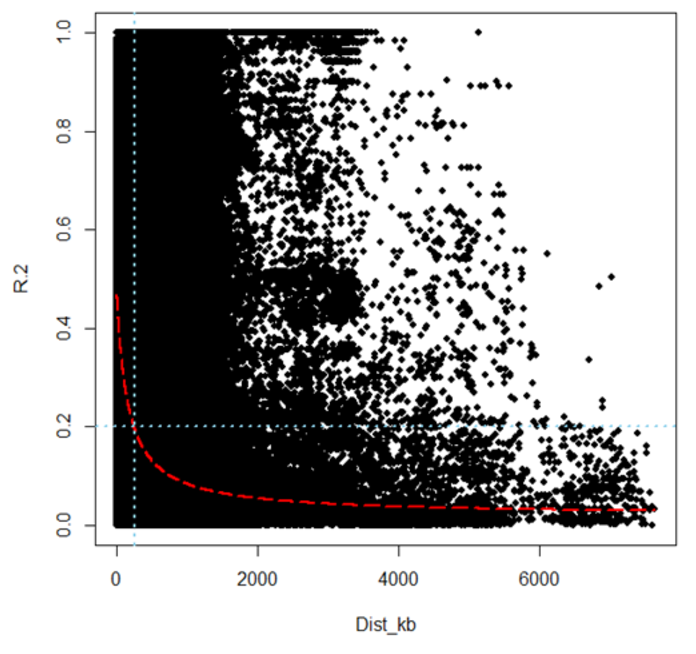

Supplement: Supplementary file 1 [file genes-13-02002-s001.zip › Figure S2.tif]

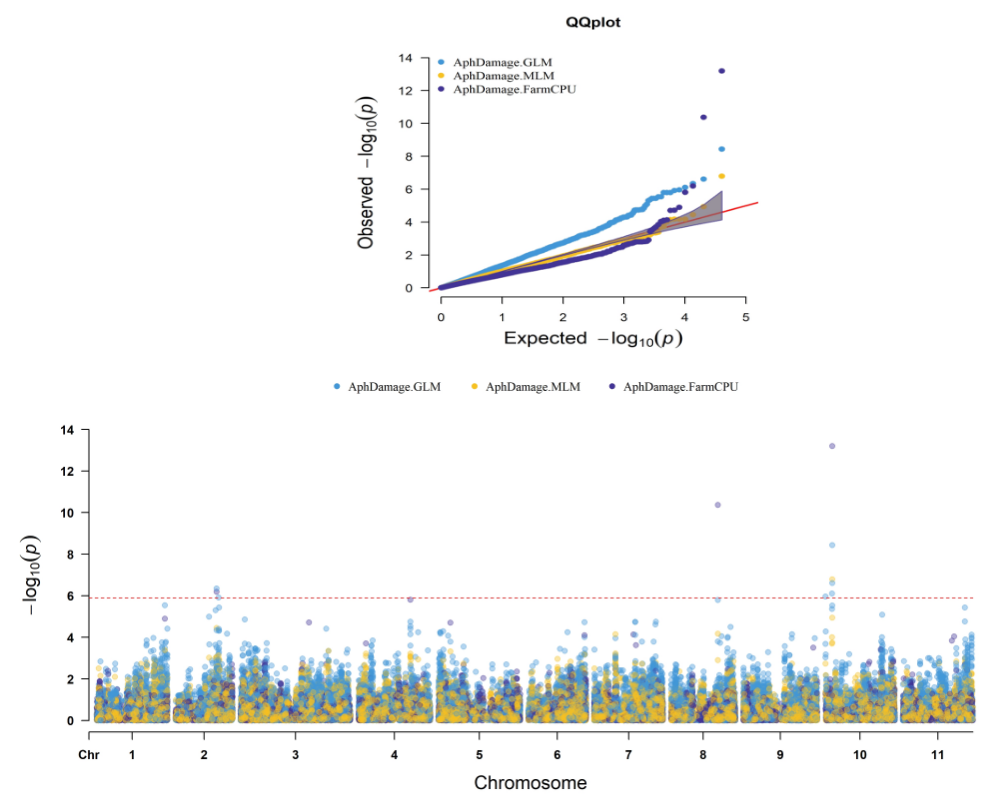

Supplement: Supplementary file 1 [file genes-13-02002-s001.zip › Figure S3.tif]
